# A wandering spleen presenting as a hypogastric mass: case report

**Published:** 2012-02-21

**Authors:** Mahdi Bouassida, Selim Sassi, Mohamed Fadhel Chtourou, Noomen Bennani, Sonia Baccari, Fathi Chebbi, Mechaal Benali, Mohamed Mongi Mighri, Hassen Touinsi, Sadok Sassi

**Affiliations:** 1 Department of surgery, Mohamed Thahar Maamouri Hospital, Nabeul, Tunisia; 2 Department of reanimation, Mohamed Thahar Maamouri Hospital, Nabeul, Tunisia

**Keywords:** Wandering spleen, torsion, CT scan, splenectomy, splenopexy

## Abstract

Wandering spleen is a rare condition characterized by the absence or underdevelopment of one or all of the ligaments that hold the spleen in its normal position in the left upper quadrant of the abdomen. It is an uncommon clinical entity that mainly affects children. Among adults it most frequently affects women of reproductive age, in whom acquired laxity of the splenic ligaments is usually the cause. Patients with a wandering spleen may be asymptomatic, present with a movable mass in the abdomen, or have chronic or intermittent abdominal pain because of partial torsion and spontaneous detorsion of the spleen. A 26-year-old woman was admitted to our hospital with vomiting and abdominal pain. Abdominal examination revealed a large ovoid hypogastric mass. A CT scan showed a wandering spleen in the hypogastric region. Exploratory laparotomy revealed an ischemic spleen. A total splenectomy was performed.

## Introduction

A wandering spleen, defined as a spleen without peritoneal attachments, is a rare entity characterized by splenic hypermobility due to laxity or maldevelopment of the supporting splenic ligaments. Patients with a wandering spleen may be asymptomatic, or may present with a palpable mass in the abdomen, or with acute, chronic, or intermittent symptoms due to torsion of the wandering spleen. The non-specific signs and symptoms together with the rarity of this condition hamper the clinical diagnosis in which imaging modalities play an important role. Treatment should be planned according to the vitality of the spleen.

### Case report

A 26-year-old woman presented to the emergency department after three days of abdominal pain, vomiting and constipation. On admission she had normal vital signs and a temperature of 38.5°C. Abdominal examination revealed marked diffuse abdominal tenderness and guarding. A large ovoid hypogastric mass was palpable. Laboratory tests revealed increased white blood cells (WBC) of 24000 µ/L. The platelets count and haemoglobin rate were normal.

**Figure 1:**
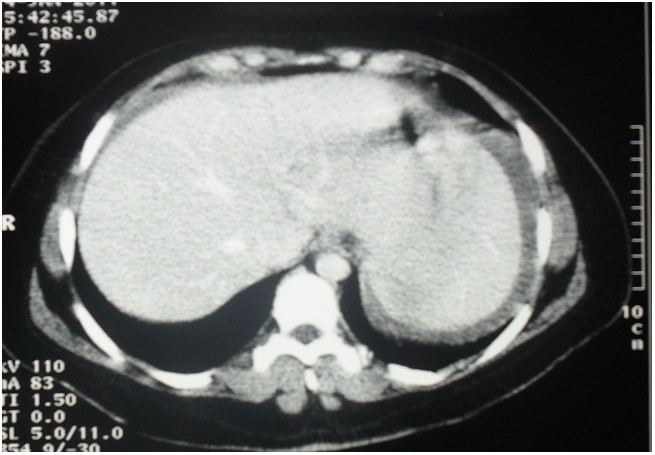
CT scan of the patient with a wandering spleen presenting as a hypogastric mass: absence of the spleen in the left upper quadrant of the abdomen

**Figure 2:**
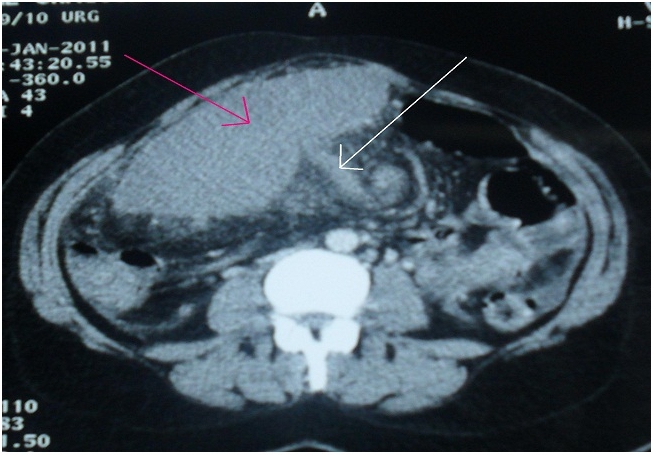
CT scan of the patient with a wandering spleen presenting as a hypogastric mass: Infracted wandering spleen (red arrow), and dilatated spleen vessels (white arrow)

A CT scan showed absence of the spleen in the left upper quadrant (Figure 1) as well as an enlarged spleen in the hypogastric region. The splenic parenchyma showed poorly, inhomogenous enhancing areas suggestive of infarction. The splenic vein was dilatated and showed a non-enhancing filling defect near the hilum, indicating the presence of a thrombosis (Figure 2).

The patient underwent exploratory laparotomy through a midline incision. This revealed the absence of all splenic ligamentous attachments and short gastric vessels with a consequent dislocation of a bigger and congested spleen in the pelvis. This organ, wrapped in the omentum, was in serious ischemic suffering due to a 240°: clock torsion around its exceptionally long pedicle (≈ 25 cm). A total splenectomy was performed. The patient′s post-operative course was uneventful. The patient was discharged with appropriate postsplenectomy treatment.

## Discussion

Wandering spleen is defined as a mobile spleen that is attached only by an elongated vascular pedicle, allowing it to migrate to any part of the abdomen or pelvis. It is a result of congenital anomalies in the development of the dorsal mesogastrium and the absence or malformation of normal splenic suspensory ligaments [1,2]. However, acquired anomalies have been described and are attributed to laxity of the ligaments due to weakness of the abdominal wall, multiple pregnancies, hormonal changes or increase in size in the spleen [3]. Both congenital and acquired conditions result in a long pedicle, which is predisposed to torsion. The splenic vessels course within the pedicle, and therefore, torsion of the pedicle results in a partial or complete infarct of the spleen [4]. Torsion of a wandering spleen is diagnosed in about 0.2-0.3% of patients who require splenectomy [5].

The clinical presentation of a wandering spleen is variable. Affected patients may be asymptomatic and this condition may be incidentally discovered on physical examination, or on imaging studies performed for other unrelated reasons, as an abdominal or pelvic mass that may not be accompanied by gastrointestinal or urinary symptoms [6]. The major complication related to splenic torsion is due to venous stasis and congestion, and splenic vein thrombosis culminating in impaired arterial supply leading to splenic infarction and necrosis. Laboratory tests are usually non-specific but may reveal elevated inflammatory markers and evidence of hypersplenism or functional asplenia [7].

Sonography showed the characteristic comma-shaped spleen in an ectopic position and the lack of splenic tissue in the left upper quadrant. Computed tomography is the preferred study for diagnosing a wandering spleen when torsion is suspected clinically or on other imaging studies.

The CT manifestations included: (1) absence of the spleen anterior to the left kidney and posterior to the stomach, (2) a lower abdominal or pelvic mass with homogenous or heterogenous splenic parenchyma and an attenuation value less than that of normal splenic tissue. Multislice spiral CT is helpful in the diagnosis at an earlier stage before the spleen progresses to infarction [8].

Detorsion and splenopexy is a reasonable surgical option, even in patients presenting with an acute abdomen, when there is no evidence of infarction, thrombosis, or hypersplenism. Splenic preservation is highly recommended in very young patients, those under 1 year of age up to those in the third decade of life, who are at particular risk for overwhelming post-splenectomy sepsis [9]. Open methods include sutured techniques with the splenic hilum fixation to the splenic bed [10], colonic displacement, the placement of the spleen in a retroperitoneal pocket (extra-peritoneal pouch) under the left costal margin [11], and the splenic snood fixation method by using absorbable mesh wrap [12] or polytetrafluoroethylene (PTFE) bridges.

Recently, laparoscopic procedures have been introduced for splenic surgery, and it has been shown to offer the benefits of minimally invasive surgery [7,9]. Laparoscopic methods include creating a pouch with natural tissue such as omentum, stomach, and colon or the use of an absorbable mesh bag to fix the spleen in its normal anatomical position. In our case, splenic preservation was not possible because of the spleen infraction, this validates any delay in diagnosis can lead to severe consequences.

## Conclusion

The diagnosis of wandering spleen is extremely difficult to establish because it is such a rare condition and is clinically nonspecific. An early diagnosis and surgical care are the best guarantee for preserving the spleen. Additional imaging examinations, especially abdominal sonogram as the imaging examination of choice, can help establish a diagnosis when faced with an abnormal location of the spleen. When wondering spleen is diagnosed, the treatment of choice is splenopexy in asymptomatic or even symptomatic patients without the presence of splenic necrosis. If splenic necrosis is present, a splenectomy usually is required.
